# Datasets on statistical analysis and performance evaluation of backtracking search optimisation algorithm compared with its counterpart algorithms

**DOI:** 10.1016/j.dib.2019.105046

**Published:** 2019-12-23

**Authors:** Bryar A. Hassan, Tarik A. Rashid

**Affiliations:** a Kurdistan Institution for Strategic Studies and Scientific Research, Sulaimani, Iraq; b Computer Science and Engineering Department, University of Kurdistan Hewler, Erbil, Iraq; c Department of Computer Networks, Technical College of Informatics, Sulaimani Polytechnic University, Sulaimani, Iraq

**Keywords:** Backtracking search optimisation algorithm, Statistical analysis, BSA experimental data, BSA performance evaluation

## Abstract

In this data article, we present the data used to evaluate the statistical success of the backtracking search optimisation algorithm (BSA) in comparison with the other four evolutionary optimisation algorithms. The data presented in this data article is related to the research article entitles ‘Operational Framework for Recent Advances in Backtracking Search Optimisation Algorithm: A Systematic Review and Performance Evaluation’ [1]. Three statistical tests conducted on BSA compared to differential evolution algorithm (DE), particle swarm optimisation (PSO), artificial bee colony (ABC), and firefly algorithm (FF). The tests are used to evaluate these mentioned algorithms and to determine which one could solve a specific optimisation problem concerning the statistical success of 16 benchmark problems taking several criteria into account. The criteria are initializing control parameters, dimension of the problems, their search space, and number of iterations needed to minimise a problem, the performance of the computer used to code the algorithms and their programming style, getting a balance on the effect of randomization, and the use of different type of optimisation problem in terms of hardness and their cohort. In addition, all the three tests include necessary statistical measures (Mean: mean-solution, S.D.: standard-deviation of mean-solution, Best: the best solution, Worst: the worst solution, Exec. Time: mean runtime in seconds, No. of succeeds: number of successful minimisation, and No. of Failure: number of failed minimisation).

**Table:** Specifications table

Subject	Theoretical Computer Science
Specific subject area	Evolutionary computation
Type of data	Table Graph
How data were acquired	Instruments: software, program
Data format	Raw
Parameters for data collection	Basic statistical measures (Mean: mean-solution, S.D.: standard-deviation of mean-solution, Best: the best solution, Worst: the worst solution, Exec. Time: mean runtime in seconds, No. of succeeds: number of successful minimisation, and No. of Failure: number of failed minimisation) of the 30-solutions obtained by BSA, DE, PSO, ABC, and FF algorithms in three tests.
Description of data collection	The data was collected from the MATLAB simulations of running five optimisation algorithms for minimising sixteen benchmark problems. The algorithms are BSA, DE, PSO, ABC, and FF. Each of these algorithms was run thirty times on each of the sixteen benchmarking problems to evaluate the performance of each of them. The collected data are presented on Mendeley Data: Hassan, Bryar; Rashid, Tarik (2019), “Data for statistical analysis and performance evaluation of backtracking search optimisation algorithm compared with its competitive algorithms”, Mendeley Data, v3 https://doi.org/10.17632/hx8xbyjmf5.3
Data source location	Institution: Kurdistan Institution for Strategic Studies and Scientific Research City/Town/Region: Sulaimani Country: Iraq Latitude and longitude (and GPS coordinates) for collected samples/data: 35.521700, 45.466605
Data accessibility	Raw/primary data are available on Mendeley Data: Hassan, Bryar; Rashid, Tarik (2019), “Data for: statistical analysis and performance evaluation of backtracking search optimisation algorithm compared with its competitive algorithms”, Mendeley Data, v3 https://doi.org/10.17632/hx8xbyjmf5.3
Related research article	B.A. Hassan, T.A. Rashid, Operational framework for recent advances in backtracking search optimisation algorithm: A systematic review and performance evaluation, Appl. Math. Comput. (2019) 124919., DOI: https://doi.org/10.1016/j.amc.2019.124919 [1]

**Table:** 

**Value of the Data** • The statistical analysis data on of backtracking search optimisation algorithm with its competitive algorithms may be useful for the researcher to better adapt these techniques for particular problems. • This dataset shows the best parameter settings used in the tests, which aid scholars to enhance reanalysis or/and reproducibility. • This dataset helps better understanding the BSA algorithm in depth by providing experiments and additional information. • This data provided gives an insight into BSA statistical performance compared with its competitors to encourage further researches on BSA in real-world applications. • Three tests on sixteen benchmark functions are used to statistically evaluate the performance of BSA compared to DE, PSO, ABS, and FF. • Necessary statistical measures are obtained by BSA, DE, PSO, ABC, and FF algorithms in three tests for sixteen benchmark functions.

## 1 Data

The dataset contains the simulation result of performance evaluation of BSA compared to its counterpart algorithms (DE, PSO, ABS, and FF). The performance of these algorithms is evaluated on minimising sixteen benchmark functions. This evaluation is conducted by three tests. The optimisation benchmark problems used in these tests are presented in Table 1. The data files (xlsx format) are the raw data results from Tests 1, 2, and 3. Additionally, information about the Tests 1, 2, and 3 are depicted in the tables as follows: (i) Table 2, Table 3, Table 4 present basic statistics of 30-solutions obtained by the algorithms in Test 1. (ii) Table 5, Table 6, Table 7 present basic statistics of 30-solutions obtained by the algorithms in Test 2. (iii) Table 8, Table 9 presents the ratio of successful minimisation of the functions for Tests 1 and 2.

**Table 1:** The sixteen benchmark problems.

Name	Success rate (%)
Ackley (F1)	48.25
Alpine01 (F2)	65.17
Bird (F3)	59.00
Leon (F4)	41.17
CrossInTray (F5)	74.08
Easom (F6)	26.08
Whitley (F7)	4.92
EggCrate (F8)	64.92
Griewank (F9)	6.08
HolderTable (F10)	80.08
Rastrigin (F11)	39.50
Rosenbrock (F12)	44.17
Salomon (F13)	10.33
Sphere (F14)	82.75
StyblinskiTang (F15)	70.50
Schwefel26 (F16)	62.67

**Table 2:** Basic statistics of the 30-solutions obtained by BSA, DE, PSO, ABC, and FF in Test 1 (default search space with Nvar1 dimensions).

Functions	Statistics	BSA	DE	PSO	ABC	FF
F1	Mean	688.3666667	209.8	NC	NC	323.3333333
	S.D.	61.19808614	5.761944116	NC	NC	7.716231586
	Best	584	193	NC	NC	301
	Worst	830	220	NC	NC	334
	Exec. time	0.3129113	0.767031967	NC	NC	8.876311833
	No. of Succeeds	30	30	0	0	30
	No. of Failures	0	0	30	30	0
F2	Mean	78.96666667	305.1666667	NC	NC	93.43333333
	S.D.	11.21723838	22.1672068	NC	NC	78.09882481
	Best	63	245	NC	NC	12
	Worst	109	348	NC	NC	248
	Exec. time	0.079013967	0.613814533	NC	NC	1.1518529
	No. of Succeeds	30	30	0	0	30
	No. of Failures	0	0	30	30	0
F3	Mean	288.2	128.7666667	54.59259259	35.33333333	32.5
	S.D.	57.68487521	40.0548618	4.901014489	7.169539654	14.28708122
	Best	193	72	44	23	6
	Worst	387	201	62	53	63
	Exec. time	0.121238167	0.392393267	0.097268	0.087277867	0.656926867
	No. of Succeeds	30	30	27	30	30
	No. of Failures	0	0	3	0	0
F4	Mean	387.7	3960.133333	857	128.3333333	1480.666667
	S.D.	127.0707514	244.6563717	675.7609249	136.9530756	34.20963096
	Best	47	3546	5	26	1388
	Worst	588	4472	1747	641	1520
	Exec. time	0.142008467	10.51661843	1.519789933	0.217805708	22.88727067
	No. of Succeeds	30	30	30	24	30
	No. of Failures	0	0	0	6	0
F5	Mean	121.1666667	38.26666667	27.23333333	14.76666667	3.766666667
	S.D.	34.72362143	8.337424283	9.814427569	4.031627834	0.678910554
	Best	45	23	6	7	2
	Worst	175	52	40	24	5
	Exec. time	0.027849683	0.095730833	0.033622767	0.042479367	0.057955067
	No. of Succeeds	30	30	30	30	30
	No. of Failures	0	0	0	0	0
F6	Mean	760.4333333	208.5	63.83333333	NC	128.1666667
	S.D.	63.97665702	33.34847357	3.939922398	NC	20.77977022
	Best	617	162	54	NC	91
	Worst	880	326	71	NC	166
	Exec. time	0.251127833	0.5434189	0.094766533	NC	1.785906667
	No. of Succeeds	30	30	30	0	30
	No. of Failures	0	0	0	30	0
F7	Mean	972	4809.6	NC	NC	NC
	S.D.	349.46797	1851.094463	NC	NC	NC
	Best	555	1616	NC	NC	NC
	Worst	1926	8339	NC	NC	NC
	Exec. time	0.505736867	8.1352712	NC	NC	NC
	No. of Succeeds	30	10	0	0	0
	No. of Failures	0	20	30	30	30
F8	Mean	119.1	2897.133333	637.5666667	15.875	5.071428571
	S.D.	30.35121994	22.93278534	4.636313438	2.997281377	1.741190981
	Best	63	2855	627	11	3
	Worst	169	2944	650	21	10
	Exec. time	0.044835	4.779958067	1.223019467	0.029835208	0.066557357
	No. of Succeeds	30	30	30	24	28
	No. of Failures	0	0	0	6	2
F9	Mean	373.1	805.2666667	NC	NC	NC
	S.D.	82.92345868	100.8679346	NC	NC	NC
	Best	285	631	NC	NC	NC
	Worst	611	1081	NC	NC	NC
	Exec. time	0.172796067	1.5184459	NC	NC	NC
	No. of Succeeds	30	30	0	0	0
	No. of Failures	0	0	30	30	30
F10	Mean	314.1333333	72.83333333	54.26666667	NC	20.83333333
	S.D.	61.64119387	14.48681525	7.315233672	NC	11.21293383
	Best	209	43	40	NC	5
	Worst	418	111	65	NC	44
	Exec. time	0.1276912	0.181376167	0.088567367	NC	0.2915058
	No. of Succeeds	30	30	30	0	30
	No. of Failures	0	0	0	30	0
F11	Mean	1038.933333	562.3666667	NC	NC	NC
	S.D.	128.4778079	28.48046316	NC	NC	NC
	Best	811	508	NC	NC	NC
	Worst	1293	634	NC	NC	NC
	Exec. time	0.379444367	0.744079967	NC	NC	NC
	No. of Succeeds	30	30	0	0	0
	No. of Failures	0	0	30	30	30
F12	Mean	1119.566667	NC	NC	NC	NC
	S.D.	307.9534989	NC	NC	NC	NC
	Best	578	NC	NC	NC	NC
	Worst	2001	NC	NC	NC	NC
	Exec. time	0.3752452	NC	NC	NC	NC
	No. of Succeeds	30	0	0	0	0
	No. of Failures	0	30	30	30	30
F13	Mean	NC	NC	NC	NC	NC
	S.D.	NC	NC	NC	NC	NC
	Best	NC	NC	NC	NC	NC
	Worst	NC	NC	NC	NC	NC
	Exec. time	NC	NC	NC	NC	NC
	No. of Succeeds	0	0	0	0	0
	No. of Failures	30	30	30	30	30
F14	Mean	151.9	154.6	1967.9	NC	12.53333333
	S.D.	31.17840765	4.123941871	517.0026579	NC	1.008013866
	Best	107	148	1782	NC	11
	Worst	208	162	4691	NC	15
	Exec. time	0.092171037	0.463599767	3.315528667	NC	0.156159333
	No. of Succeeds	30	30	30	0	30
	No. of Failures	0	0	0	30	0
F15	Mean	490.7666667	NC	100	659.6666667	245.8461538
	S.D.	56.80073053	NC	5.253570215	108.7443485	4.017589531
	Best	345	NC	94	516	240
	Worst	598	NC	109	949	253
	Exec. time	0.2717145	NC	0.188506	1.422079033	3.067869077
	No. of Succeeds	30	0	11	30	13
	No. of Failures	0	30	19	0	17
F16	Mean	NC	254.9615385	NC	NC	NC
	S.D.	NC	14.32614608	NC	NC	NC
	Best	NC	231	NC	NC	NC
	Worst	NC	289	NC	NC	NC
	Exec. time	NC	0.572146308	NC	NC	NC
	No. of Succeeds	0	26	0	0	0
	No. of Failures	30	4	30	30	30

**Table 3:** Basic statistics of the 30-solutions obtained by BSA, DE, PSO, ABC, and FF in Test 1 (default search space with Nvar2 dimensions).

Functions	Statistics	BSA	DE	PSO	ABC	FF
F1	Mean	1391.866667	600.8	NC	NC	373.0333333
	S.D.	240.1736058	11.12747454	NC	NC	4.31903033
	Best	949	572	NC	NC	362
	Worst	1899	626	NC	NC	381
	Exec. time	0.546482767	2.1215337	NC	NC	9.566863367
	No. of Succeeds	30	30	0	0	30
	No. of Failures	0	0	30	30	0
F2	Mean	467.4333333	2653.133333	NC	NC	346.0333333
	S.D.	54.7487574	311.8812477	NC	NC	34.27121375
	Best	397	2136	NC	NC	227
	Worst	601	3367	NC	NC	408
	Exec. time	0.2806565	6.5514224	NC	NC	6.9452777
	No. of Succeeds	30	30	0	0	30
	No. of Failures	0	0	30	30	0
F3	Mean	346.6333333	170.8666667	60.79310345	35.6	32.7
	S.D.	84.07528783	38.91346131	28.86592073	6.003447286	16.26748396
	Best	182	99	44	26	5
	Worst	505	236	209	45	67
	Exec. time	0.190157233	0.5601642	0.115230897	0.0631293	0.644730733
	No. of Succeeds	30	30	29	30	30
	No. of Failures	0	0	1	0	0
F4	Mean	471.6333333	5530.1	1152.866667	91.71428571	1492
	S.D.	139.2421566	266.6691544	496.0978583	41.26953602	38.38821658
	Best	180	4762	4	23	1323
	Worst	862	6071	1644	160	1533
	Exec. time	0.193297133	14.77711263	2.1152108	0.253110071	22.1456451
	No. of Succeeds	30	30	30	28	30
	No. of Failures	0	0	0	2	0
F5	Mean	169.1	41.76666667	29.86666667	13.8	3.733333333
	S.D.	49.42800407	11.49717607	11.64335931	4.604345773	0.868344971
	Best	70	14	2	2	2
	Worst	248	62	54	22	5
	Exec. time	0.044610567	0.106829767	0.046657733	0.044076667	0.051693733
	No. of Succeeds	30	30	30	30	30
	No. of Failures	0	0	0	0	0
F6	Mean	912.9	267.5666667	64.8	NC	121.7333333
	S.D.	97.77713928	41.86487568	4.574290976	NC	34.65985786
	Best	678	194	55	NC	19
	Worst	1045	386	75	NC	173
	Exec. time	0.318349367	0.724312633	0.127859867	NC	1.7146897
	No. of Succeeds	30	30	30	0	30
	No. of Failures	0	0	0	30	0
F7	Mean	4764.566667	NC	NC	NC	NC
	S.D.	624.9610572	NC	NC	NC	NC
	Best	3887	NC	NC	NC	NC
	Worst	6810	NC	NC	NC	NC
	Exec. time	3.934701977	NC	NC	NC	NC
	No. of Succeeds	30	0	0	0	0
	No. of Failures	0	30	30	30	30
F8	Mean	133.1666667	3542.366667	637.3333333	15.55172414	4.666666667
	S.D.	35.99337423	39.65409344	5.441433212	2.599260979	1.109400392
	Best	35	3436	625	9	3
	Worst	203	3610	651	21	8
	Exec. time	0.063654233	6.444122133	1.30194	0.030338172	0.077771407
	No. of Succeeds	30	30	30	29	27
	No. of Failures	0	0	0	1	3
F9	Mean	587.0333333	1297	NC	NC	NC
	S.D.	80.61123535	45.5290737	NC	NC	NC
	Best	469	1234	NC	NC	NC
	Worst	841	1423	NC	NC	NC
	Exec. time	0.295489567	2.409856833	NC	NC	NC
	No. of Succeeds	30	30	0	0	0
	No. of Failures	0	0	30	30	30
F10	Mean	366.8666667	91.63333333	199.1666667	NC	19.8
	S.D.	42.6790212	14.87994098	554.8413929	NC	10.03579799
	Best	301	63	36	NC	5
	Worst	457	125	2450	NC	45
	Exec. time	0.187796133	0.2636237	0.357409	NC	0.275910433
	No. of Succeeds	30	30	30	0	30
	No. of Failures	0	0	0	30	0
F11	Mean	3761.9	4211.533333	NC	NC	NC
	S.D.	503.0939687	835.0827642	NC	NC	NC
	Best	2890	3339	NC	NC	NC
	Worst	4679	7001	NC	NC	NC
	Exec. time	1.841760067	6.437364733	NC	NC	NC
	No. of Succeeds	30	30	0	0	0
	No. of Failures	0	0	30	30	30
F12	Mean	1518.7	NC	NC	NC	NC
	S.D.	520.5891987	NC	NC	NC	NC
	Best	871	NC	NC	NC	NC
	Worst	2901	NC	NC	NC	NC
	Exec. time	0.457080213	NC	NC	NC	NC
	No. of Succeeds	30	0	0	0	0
	No. of Failures	0	30	30	30	30
F13	Mean	NC	NC	NC	NC	NC
	S.D.	NC	NC	NC	NC	NC
	Best	NC	NC	NC	NC	NC
	Worst	NC	NC	NC	NC	NC
	Exec. time	NC	NC	NC	NC	NC
	No. of Succeeds	0	0	0	0	0
	No. of Failures	30	30	30	30	30
F14	Mean	417	484.4333333	NC	714.3	54.36666667
	S.D.	56.51914537	7.219147592	NC	77.84739714	2.85854235
	Best	303	469	NC	568	50
	Worst	531	497	NC	834	63
	Exec. time	0.2572861	0.841805833	NC	2.2013179	0.5315005
	No. of Succeeds	30	30	0	30	30
	No. of Failures	0	0	30	0	0
F15	Mean	NC	NC	NC	NC	NC
	S.D.	NC	NC	NC	NC	NC
	Best	NC	NC	NC	NC	NC
	Worst	NC	NC	NC	NC	NC
	Exec. time	NC	NC	NC	NC	NC
	No. of Succeeds	0	0	0	0	0
	No. of Failures	30	30	30	30	30
F16	Mean	NC	991	NC	NC	NC
	S.D.	NC	1078.5	NC	NC	NC
	Best	NC	52.59882474	NC	NC	NC
	Worst	NC	991	NC	NC	NC
	Exec. time	NC	1200	NC	NC	NC
	No. of Succeeds	0	12	0	0	0
	No. of Failures	30	18	30	30	30

**Table 4:** Basic statistics of the 30-solutions obtained by BSA, DE, PSO, ABC, and FF in Test 1 (default search space with Nvar3 dimensions).

Functions	Statistics	BSA	DE	PSO	ABC	FF
F1	Mean	2212.033333	1265.4	NC	NC	409.8
	S.D.	285.8103402	17.29380994	NC	NC	2.578425074
	Best	1789	1231	NC	NC	404
	Worst	2705	1297	NC	NC	414
	Exec. time	1.2222052	4.161145333	NC	NC	6.678406533
	No. of Succeeds	30	30	0	0	30
	No. of Failures	0	0	30	30	0
F2	Mean	1437.033333	1233.333333	NC	NC	453.4666667
	S.D.	192.1665017	188.0521449	NC	NC	26.07381211
	Best	1129	907	NC	NC	411
	Worst	1982	1619	NC	NC	542
	Exec. time	0.946699633	2.589517933	NC	NC	9.553941067
	No. of Succeeds	30	30	0	0	30
	No. of Failures	0	0	30	30	0
F3	Mean	364.7666667	186.8666667	53.80769231	38.33333333	28.26666667
	S.D.	73.52535726	51.15475731	5.592990118	8.326663998	19.5711493
	Best	242	124	39	21	4
	Worst	525	284	61	56	81
	Exec. time	0.217162567	0.4063774	0.105839692	0.0705598	0.603975867
	No. of Succeeds	30	30	26	30	30
	No. of Failures	0	0	4	0	0
F4	Mean	525.7	6225.6	909.4	84.7037037	1489.633333
	S.D.	218.5027657	461.136198	641.2112245	43.79481425	32.82606548
	Best	284	5442	6	23	1409
	Worst	1269	7563	1837	186	1531
	Exec. time	0.2490285	14.56781393	2.5651712	0.266280556	20.34113953
	No. of Succeeds	30	30	30	27	30
	No. of Failures	0	0	0	3	0
F5	Mean	173.5333333	45.3	24.2	14.53333333	3.666666667
	S.D.	39.28299916	10.16807038	10.69772776	4.904138529	0.884086645
	Best	82	25	6	2	2
	Worst	234	64	41	23	5
	Exec. time	0.109724933	0.132266967	0.043759533	0.045434933	0.051644167
	No. of Succeeds	30	30	30	30	30
	No. of Failures	0	0	0	0	0
F6	Mean	996.1	296.5666667	64.16666667	NC	113.7333333
	S.D.	88.20757259	47.62691454	4.609460536	NC	26.92509832
	Best	861	209	55	NC	56
	Worst	1197	408	76	NC	168
	Exec. time	0.4946741	0.879912567	0.118392067	NC	1.790483167
	No. of Succeeds	30	30	30	0	30
	No. of Failures	0	0	0	30	0
F7	Mean	NC	NC	NC	NC	NC
	S.D.	NC	NC	NC	NC	NC
	Best	NC	NC	NC	NC	NC
	Worst	NC	NC	NC	NC	NC
	Exec. time	NC	NC	NC	NC	NC
	No. of Succeeds	0	0	0	0	0
	No. of Failures	30	30	30	30	30
F8	Mean	118.2	3750.9	637.0333333	15.03571429	4.5
	S.D.	27.9425519	44.63055239	6.392254652	2.486737307	0.989949494
	Best	78	3643	624	10	2
	Worst	190	3811	649	20	6
	Exec. time	0.169004103	6.867129	1.4425986	0.029832	0.073269769
	No. of Succeeds	30	30	30	28	26
	No. of Failures	0	0	0	2	4
F9	Mean	965.9666667	2588.033333	NC	NC	NC
	S.D.	76.88974791	72.99715787	NC	NC	NC
	Best	855	2514	NC	NC	NC
	Worst	1150	2898	NC	NC	NC
	Exec. time	0.582512467	5.4244718	NC	NC	NC
	No. of Succeeds	30	30	0	0	0
	No. of Failures	0	0	30	30	30
F10	Mean	397.2333333	92.83333333	56.66666667	NC	20.83333333
	S.D.	43.98172295	15.20681184	5.168427583	NC	10.19493902
	Best	309	41	49	NC	7
	Worst	471	114	67	NC	38
	Exec. time	9565.148219	0.241654933	0.099469833	NC	0.284334333
	No. of Succeeds	30	30	30	0	30
	No. of Failures	0	0	0	30	0
F11	Mean	NC	NC	NC	NC	NC
	S.D.	NC	NC	NC	NC	NC
	Best	NC	NC	NC	NC	NC
	Worst	NC	NC	NC	NC	NC
	Exec. time	NC	NC	NC	NC	NC
	No. of Succeeds	0	0	0	0	0
	No. of Failures	30	30	30	30	30
F12	Mean	16054.2	NC	NC	NC	NC
	S.D.	22085.31046	NC	NC	NC	NC
	Best	9065	NC	NC	NC	NC
	Worst	132801	NC	NC	NC	NC
	Exec. time	6.478484033	NC	NC	NC	NC
	No. of Succeeds	30	0	0	0	0
	No. of Failures	0	30	30	30	30
F13	Mean	NC	NC	NC	NC	NC
	S.D.	NC	NC	NC	NC	NC
	Best	NC	NC	NC	NC	NC
	Worst	NC	NC	NC	NC	NC
	Exec. time	NC	NC	NC	NC	NC
	No. of Succeeds	0	0	0	0	0
	No. of Failures	30	30	30	30	30
F14	Mean	943.9	1060	NC	6468.933333	113.1333333
	S.D.	84.9061754	15.59840841	NC	311.951912	3.329422994
	Best	798	1034	NC	5878	106
	Worst	1109	1097	NC	7116	120
	Exec. time	0.621603966	3.001068333	NC	19.45626283	1.025718967
	No. of Succeeds	30	30	0	30	30
	No. of Failures	0	0	30	0	0
F15	Mean	NC	NC	NC	NC	NC
	S.D.	NC	NC	NC	NC	NC
	Best	NC	NC	NC	NC	NC
	Worst	NC	NC	NC	NC	NC
	Exec. time	NC	NC	NC	NC	NC
	No. of Succeeds	0	0	0	0	0
	No. of Failures	30	30	30	30	30
F16	Mean	NC	NC	NC	NC	NC
	S.D.	NC	NC	NC	NC	NC
	Best	NC	NC	NC	NC	NC
	Worst	NC	NC	NC	NC	NC
	Exec. time	NC	NC	NC	NC	NC
	No. of Succeeds	0	0	0	0	0
	No. of Failures	30	30	30	30	30

**Table 5:** Basic statistics of the 30-solutions obtained by BSA, DE, PSO, ABC, and FF in Test 2 (two-variable dimensions with R1).

Functions	Statistics	BSA	DE	PSO	ABC	FF
F1	Mean	72.33333333	NC	0	19.33333333	60.36666667
	S.D.	16.00287331	NC	NC	2.368374	23.48217715
	Best	45	NC	NC	14	5
	Worst	101	NC	NC	23	95
	Exec. time	0.028775833	NC	NC	0.159118567	1.193958333
	No. of Succeeds	30	0	NC	30	30
	No. of Failures	0	30	0	0	0
F2	Mean	39.5	15.26666667	794.25	9.133333333	4.033333333
	S.D.	11.70838191	2.935318033	379.0831654	1.907034769	0.808716878
	Best	22	10	154	4	2
	Worst	76	22	1146	13	5
	Exec. time	0.00711624	0.045706	1.445961536	0.0771137	0.064563133
	No. of Succeeds	30	30	28	30	30
	No. of Failures	0	0	2	0	0
F3	Mean	139.5	53.33333333	53.75	NC	31.63333333
	S.D.	28.07717198	14.21347911	9.777619948	NC	12.64224809
	Best	73	32	40	NC	9
	Worst	206	79	97	NC	59
	Exec. time	0.076975133	0.084473933	0.092407821	NC	0.493045367
	No. of Succeeds	30	30	28	0	30
	No. of Failures	0	0	2	30	0
F4	Mean	377.1333333	89.69230769	1213.866667	16	9.269230769
	S.D.	68.4693404	33.18405549	159.6013856	47.125	3.053623321
	Best	286	28	926	33.18369492	5
	Worst	649	191	1550	6	19
	Exec. time	0.200669233	0.293420846	2.0382361	147	0.126300154
	No. of Succeeds	30	26	30	0.281062708	26
	No. of Failures	0	4	0	24	4
F5	Mean	45.56666667	15.03333333	21.8	7.433333333	3.133333333
	S.D.	17.02469471	5.18275212	9.459168149	3.339247627	0.730296743
	Best	13	5	3	2	2
	Worst	76	24	37	13	4
	Exec. time	0.019755067	0.032688133	0.039887567	0.077219	0.0421937
	No. of Succeeds	30	30	30	30	30
	No. of Failures	0	0	0	0	0
F6	Mean	248.2666667	30.86666667	794.25	20.3	11.96666667
	S.D.	31.22325982	4.108303898	379.0831654	1.600646421	7.716901886
	Best	202	25	154	17	4
	Worst	311	45	1146	24	34
	Exec. time	0.0888098	0.051433367	0.0724958	0.214975967	0.094171033
	No. of Succeeds	30	30	28	30	30
	No. of Failures	0	0	2	0	0
F7	Mean	64.36666667	139.5714286	NC	27.6	249.9473684
	S.D.	12.86396553	16.33997274	NC	22.41568999	26.50885321
	Best	41	103	NC	9	193
	Worst	91	170	NC	89	282
	Exec. time	0.038450533	0.427799464	NC	0.25814615	3.344931158
	No. of Succeeds	30	28	0	20	19
	No. of Failures	0	2	30	10	11
F8	Mean	248.5666667	18.48148148	636.9	12.24	4.851851852
	S.D.	23.91894839	2.359438825	5.984750737	2.241279397	0.948833442
	Best	206	15	627	8	4
	Worst	300	23	651	16	7
	Exec. time	0.042607433	0.032870519	0.713972767	0.09773984	0.068419926
	No. of Succeeds	30	27	30	25	27
	No. of Failures	0	3	0	5	3
F9	Mean	25.23333333	166.5769231	100.7142857	9.076923077	539.3793103
	S.D.	7.833100999	110.8112532	5.13552591	5.483962632	45.94671898
	Best	14	89	92	2	413
	Worst	47	691	109	19	597
	Exec. time	0.016005467	0.554092731	0.162314571	0.046853615	6.89237431
	No. of Succeeds	30	26	14	26	29
	No. of Failures	0	4	16	4	1
F10	Mean	36.9	60.15	NC	NC	5.333333333
	S.D.	17.7906407	179.1662783	NC	NC	1.688364508
	Best	16	14	NC	NC	3
	Worst	79	821	NC	NC	10
	Exec. time	0.029989533	0.17157865	NC	NC	0.084701533
	No. of Succeeds	30	20	0	0	30
	No. of Failures	0	10	30	30	0
F11	Mean	111.3666667	81.86666667	105.5	29.22222222	9.96
	S.D.	16.08486831	3.559671947	4.289120157	6.405126152	3.813135193
	Best	79	76	93	16	4
	Worst	147	89	117	45	20
	Exec. time	0.034564033	0.277437967	0.1759911	0.152298889	0.14458784
	No. of Succeeds	30	30	30	27	25
	No. of Failures	0	0	0	3	5
F12	Mean	157.9333333	60.77272727	722.8	35.88888889	8.083333333
	S.D.	35.35332334	24.92069673	104.1181027	24.95585847	2.019829237
	Best	98	8	414	5	5
	Worst	236	103	857	94	13
	Exec. time	0.145922333	0.145608045	1.219939367	0.322523741	0.111710708
	No. of Succeeds	30	22	30	27	24
	No. of Failures	0	8	0	3	6
F13	Mean	271.8	147.137931	646.36	133.7666667	10.92592593
	S.D.	40.4086027	52.0286488	5.321967055	100.097774	2.840990157
	Best	201	57	634	27	6
	Worst	344	260	656	349	17
	Exec. time	0.051252967	0.229098862	1.19053768	1.2913116	0.168615333
	No. of Succeeds	30	29	25	30	27
	No. of Failures	0	1	5	0	3
F14	Mean	232.7666667	10.2	637.5	6	2.68
	S.D.	20.00979645	2.483630619	5.544490897	1.560378995	0.476095229
	Best	190	6	626	3	2
	Worst	275	14	654	9	3
	Exec. time	0.077608533	0.034451	1.2173071	0.05095925	0.033408
	No. of Succeeds	30	20	30	24	25
	No. of Failures	0	10	0	6	5
F15	Mean	52.06666667	22.43333333	44.16666667	19.1	11.7
	S.D.	21.2244708	2.51455533	6.454527058	3.262641198	5.408486307
	Best	21	18	27	10	3
	Worst	90	27	52	25	23
	Exec. time	0.031657633	0.0592526	0.0887543	0.138674967	0.151984633
	No. of Succeeds	30	30	30	30	30
	No. of Failures	0	0	0	0	0
F16	Mean	NC	NC	NC	NC	NC
	S.D.	NC	NC	NC	NC	NC
	Best	NC	NC	NC	NC	NC
	Worst	NC	NC	NC	NC	NC
	Exec. time	NC	NC	NC	NC	NC
	No. of Succeeds	0	0	0	0	0
	No. of Failures	30	30	30	30	30

**Table 6:** Basic statistics of the 30-solutions obtained by BSA, DE, PSO, ABC, and FF in Test 2 (two variable dimensions with R2).

Functions	Statistics	BSA	DE	PSO	ABC	FF
F1	Mean	425.7333333	NC	NC	NC	249.4137931
	S.D.	65.63111822	NC	NC	NC	24.0735736
	Best	338	NC	NC	NC	192
	Worst	535	NC	NC	NC	295
	Exec. time	0.129019233	NC	NC	NC	5.009911103
	No. of Succeeds	30	0	0	0	29
	No. of Failures	0	30	30	30	1
F2	Mean	156.0333333	39.10344828	732.0740741	38.33333333	61
	S.D.	16.09558374	5.1084302	407.9909431	6.199740447	22.28486896
	Best	126	26	166	27	11
	Worst	188	49	1158	49	93
	Exec. time	0.039772367	0.112101655	1.457569407	0.197538433	0.884010333
	No. of Succeeds	30	29	27	30	27
	No. of Failures	0	1	3	0	3
F3	Mean	1087.666667	233.3888889	81.73333333	NC	208.0333333
	S.D.	391.2479885	71.84144215	6.356822744	NC	33.0146084
	Best	293	103	72	NC	117
	Worst	1719	376	97	NC	250
	Exec. time	0.1936562	0.3856845	0.1286414	NC	3.080032433
	No. of Succeeds	30	18	30	0	30
	No. of Failures	0	12	0	30	0
F4	Mean	419.7666667	194.0714286	1253.52381	219.6896552	93.8
	S.D.	191.3771657	65.9005584	154.2182282	132.8401573	24.72515588
	Best	189	64	981	33	47
	Worst	898	367	1517	636	145
	Exec. time	0.263658433	0.674849321	2.085545905	1.637981655	1.34841468
	No. of Succeeds	30	28	21	29	25
	No. of Failures	0	2	9	1	5
F5	Mean	99.93333333	29	54	NC	40.3
	S.D.	22.2539161	3.695290572	10.22168081	NC	26.1786304
	Best	53	22	28	NC	3
	Worst	143	36	68	NC	85
	Exec. time	0.043615233	0.0518579	0.0923664	NC	0.349824067
	No. of Succeeds	30	30	30	0	30
	No. of Failures	0	0	0	30	0
F6	Mean	671.8	230.1333333	75.13333333	NC	NC
	S.D.	107.4107167	48.5107406	5.888231005	NC	NC
	Best	424	162	61	NC	NC
	Worst	883	341	91	NC	NC
	Exec. time	0.229059867	0.646945533	0.120696033	NC	NC
	No. of Succeeds	30	30	30	0	0
	No. of Failures	0	0	0	30	30
F7	Mean	112.6333333	157.6551724	NC	30.61111111	445.8666667
	S.D.	30.11527661	16.00061575	NC	9.798792776	33.75033418
	Best	84	125	NC	16	343
	Worst	232	186	NC	58	499
	Exec. time	0.0441853	0.534595448	NC	0.272917111	5.854646267
	No. of Succeeds	30	29	0	18	30
	No. of Failures	0	1	30	12	0
F8	Mean	328.6666667	32.08	644.2333333	23.76	110.7666667
	S.D.	31.07425293	3.414674216	5.992428172	3.3326666	24.39712156
	Best	261	24	633	17	53
	Worst	419	37	661	29	151
	Exec. time	0.0566179	0.05600776	1.142361633	0.18997352	1.5272697
	No. of Succeeds	30	25	30	25	30
	No. of Failures	0	5	0	5	0
F9	Mean	181.5333333	175.8	794.25	201.5769231	742.3103448
	S.D.	44.83743305	19.91256751	379.0831654	144.6715378	47.85178266
	Best	104	145	154	32	547
	Worst	262	213	1146	488	807
	Exec. time	0.1036287	0.459873733	0.419778435	0.98108872	9.115128828
	No. of Succeeds	30	30	28	26	29
	No. of Failures	0	0	2	4	1
F10	Mean	NC	NC	NC	NC	NC
	S.D.	NC	NC	NC	NC	NC
	Best	NC	NC	NC	NC	NC
	Worst	NC	NC	NC	NC	NC
	Exec. time	NC	NC	NC	NC	NC
	No. of Succeeds	0	0	0	0	0
	No. of Failures	30	30	30	30	30
F11	Mean	138.1666667	96.83333333	122.4827586	43	149.8148148
	S.D.	28.06652606	4.177801215	5.925855512	7.541883054	38.67421824
	Best	94	85	114	31	50
	Worst	201	103	142	55	210
	Exec. time	0.059349433	0.282379867	0.199407138	0.424502846	2.34202537
	No. of Succeeds	30	30	29	26	27
	No. of Failures	0	0	1	4	3
F12	Mean	572.8333333	368.2222222	936.1	409.5882353	80.37037037
	S.D.	122.3739558	99.16549234	243.0311957	225.0005719	27.80999841
	Best	411	210	605	55	19
	Worst	934	565	1858	944	120
	Exec. time	0.310757733	1.056444556	1.5107343	3.453786647	1.234736259
	No. of Succeeds	30	27	30	17	27
	No. of Failures	0	3	0	13	3
F13	Mean	427.8666667	179.8333333	764.962963	204.9259259	118.4333333
	S.D.	68.73172902	39.81689412	229.5504774	118.616034	25.96374572
	Best	311	80	647	23	56
	Worst	601	270	1392	457	163
	Exec. time	0.123285933	0.2651661	1.240302815	1.956210444	1.893014833
	No. of Succeeds	30	30	27	27	30
	No. of Failures	0	0	3	3	0
F14	Mean	269.0666667	23.85185185	642.8333333	17.16666667	41.35714286
	S.D.	29.88706713	3.134224278	6.772578246	1.340560125	15.61626475
	Best	206	18	630	15	14
	Worst	339	29	658	20	74
	Exec. time	0.0919827	0.066911815	1.159890367	0.1416615	0.626989893
	No. of Succeeds	30	27	30	24	28
	No. of Failures	0	3	0	6	2
F15	Mean	135.0333333	36.86666667	68.33333333	31.33333333	163.8
	S.D.	27.06758251	3.702127378	3.950847428	3.283536081	25.20180618
	Best	85	27	58	24	87
	Worst	233	44	74	38	207
	Exec. time	0.033026633	0.087536133	0.1189461	0.236676	1.600936967
	No. of Succeeds	30	30	30	30	30
	No. of Failures	0	0	0	0	0
F16	Mean	NC	NC	NC	NC	NC
	S.D.	NC	NC	NC	NC	NC
	Best	NC	NC	NC	NC	NC
	Worst	NC	NC	NC	NC	NC
	Exec. time	NC	NC	NC	NC	NC
	No. of Succeeds	0	0	0	0	0
	No. of Failures	30	30	30	30	30

**Table 7:** Basic statistics of the 30-solutions obtained by BSA, DE, PSO, ABC, and FF in Test 2 (two-variable dimensions with R3).

Functions	Statistics	BSA	DE	PSO	ABC	FF
F1	Mean	556.6333333	NC	NC	NC	279.75
	S.D.	112.7296073	NC	NC	NC	33.31962437
	Best	396	NC	NC	NC	171
	Worst	898	NC	NC	NC	315
	Exec. time	0.189253867	NC	NC	NC	5.1484892
	No. of Succeeds	30	0	0	0	20
	No. of Failures	0	30	30	30	10
F2	Mean	154.6666667	46.16666667	847.8571429	47.46666667	88.46666667
	S.D.	24.94730078	6.411645296	367.6544991	12.19308264	26.17017694
	Best	113	27	165	32	36
	Worst	201	63	1152	95	141
	Exec. time	0.067592267	0.139866267	1.5282565	0.340741933	1.4476881
	No. of Succeeds	30	30	28	30	30
	No. of Failures	0	0	2	0	0
F3	Mean	1909.433333	182.75	84.46666667	NC	249.6
	S.D.	591.0772564	82.99949799	10.78547341	NC	29.66897834
	Best	1402	112	67	NC	159
	Worst	3725	302	118	NC	294
	Exec. time	0.3167525	0.139866267	0.127532267	NC	3.4745561
	No. of Succeeds	30	4	30	0	30
	No. of Failures	0	26	0	30	0
F4	Mean	444.0666667	220.0357143	1482.153846	238.2592593	118.6153846
	S.D.	81.262	62.51487654	824.6896479	120.881249	31.97258441
	Best	309	100	955	45	51
	Worst	659	365	5332	415	180
	Exec. time	0.179457933	0.7116585	1.445961536	2.140455593	1.692470923
	No. of Succeeds	30	28	26	27	26
	No. of Failures	0	2	4	3	4
F5	Mean	NC	NC	NC	NC	NC
	S.D.	NC	NC	NC	NC	NC
	Best	NC	NC	NC	NC	NC
	Worst	NC	NC	NC	NC	NC
	Exec. time	NC	NC	NC	NC	NC
	No. of Succeeds	0	0	0	0	0
	No. of Failures	30	30	30	30	30
F6	Mean	1096.466667	570.2666667	82.76666667	NC	NC
	S.D.	263.5093054	179.2411078	8.071590594	NC	NC
	Best	691	295	66	NC	NC
	Worst	1590	984	100	NC	NC
	Exec. time	0.3102513	1.701697967	0.111516034	NC	NC
	No. of Succeeds	30	30	30	0	0
	No. of Failures	0	0	0	30	30
F7	Mean	165.1333333	158.3103448	NC	36.83333333	474.3
	S.D.	44.85281165	18.3655412	NC	20.98809186	31.4896261
	Best	98	117	NC	22	409
	Worst	292	188	NC	116	528
	Exec. time	0.107162467	0.423307759	NC	0.336140889	6.128933267
	No. of Succeeds	30	29	0	18	30
	No. of Failures	0	1	30	12	0
F8	Mean	319.0666667	35.30769231	643.8333333	27.2	145.3043478
	S.D.	38.74935669	2.412786452	6.198349799	2.645751311	19.00104012
	Best	271	31	634	22	115
	Worst	401	40	657	31	182
	Exec. time	0.0543112	0.060988577	1.248304667	0.21520208	2.085555391
	No. of Succeeds	30	26	30	25	23
	No. of Failures	0	4	0	5	7
F9	Mean	145.1666667	176.3	370.7083333	179.9615385	789.7666667
	S.D.	36.97257449	17.78831465	400.0615691	118.135001	27.78945885
	Best	99	147	118	29	725
	Worst	254	207	1277	460	842
	Exec. time	0.08441	0.555940233	0.588364917	0.943245115	8.9030898
	No. of Succeeds	30	30	24	26	30
	No. of Failures	0	0	6	4	0
F10	Mean	NC	NC	NC	NC	NC
	S.D.	NC	NC	NC	NC	NC
	Best	NC	NC	NC	NC	NC
	Worst	NC	NC	NC	NC	NC
	Exec. time	NC	NC	NC	NC	NC
	No. of Succeeds	0	0	0	0	0
	No. of Failures	30	30	30	30	30
F11	Mean	143.9333333	98.93333333	129.9333333	45.42857143	195.5517241
	S.D.	38.00901285	4.555658349	19.80061534	7.593508785	30.62513698
	Best	89	88	115	25	91
	Worst	211	108	227	60	242
	Exec. time	0.1755195	0.305618167	0.2071013	0.423829429	3.17409831
	No. of Succeeds	30	30	30	28	29
	No. of Failures	0	0	0	2	1
F12	Mean	1379.166667	427.9230769	898.9	569.5714286	116.5
	S.D.	156.3588012	117.8514058	254.7484696	246.4879018	38.30820432
	Best	1101	263	585	342	24
	Worst	1879	667	1753	932	181
	Exec. time	0.4534084	0.7652505	1.454673867	4.816096714	1.725299179
	No. of Succeeds	30	26	30	7	28
	No. of Failures	0	4	0	23	2
F13	Mean	381.5666667	171.5333333	700.1785714	224.8076923	154.1
	S.D.	102.9180066	53.29147783	134.0732397	135.1523642	29.25553293
	Best	205	75	645	46	76
	Worst	756	276	1374	456	199
	Exec. time	0.1637319	0.519127567	1.213026786	2.209036731	2.474901667
	No. of Succeeds	30	30	28	26	30
	No. of Failures	0	0	2	4	0
F14	Mean	280.0333333	26.35714286	644.2333333	18.06896552	66.78571429
	S.D.	26.29210362	3.291001038	6.371722732	2.750727624	25.65635744
	Best	235	17	633	10	22
	Worst	344	31	657	21	105
	Exec. time	0.0942649	0.082852964	1.2564309	0.157990172	1.06789675
	No. of Succeeds	30	28	30	29	28
	No. of Failures	0	2	0	1	2
F15	Mean	NC	39.46666667	70.66666667	33.3	185.5666667
	S.D.	NC	2.885556581	4.309839051	3.052980454	33.59359887
	Best	NC	34	62	26	111
	Worst	NC	44	77	39	241
	Exec. time	NC	0.1049036	0.106110233	0.2379349	1.4060248
	No. of Succeeds	0	30	30	30	30
	No. of Failures	30	0	0	0	0
F16	Mean	NC	NC	NC	NC	NC
	S.D.	NC	NC	NC	NC	NC
	Best	NC	NC	NC	NC	NC
	Worst	NC	NC	NC	NC	NC
	Exec. time	NC	NC	NC	NC	NC
	No. of Succeeds	0	0	0	0	0
	No. of Failures	30	30	30	30	30

**Table 8:** The success and failure ratio for minimising the sixteen benchmark functions in Test 1.

Variable dimensions	BSA		DE		PSO		ABC		FF	
	Success	Failure	Success	Failure	Success	Failure	Success	Failure	Success	Failure
Nvar1: 10	11	2	13	3	9	7	6	10	10	6
Nvar2: 30	13	3	12	4	6	10	5	11	9	7
Nvar3: 60	11	5	10	6	6	10	5	11	9	7

**Table 9:** The success and failure ratio for minimising the sixteen benchmark functions in Test 2.

Search space	BSA		DE		PSO		ABC		FF	
	Success	Failure	Success	Failure	Success	Failure	Success	Failure	Success	Failure
R1: [-5, 5]	15	1	14	2	12	4	13	3	15	1
R2: [-250, 205]	14	2	13	3	12	4	12	4	13	3
R3: [-500, 500]	12	4	12	4	10	6	10	6	12	4

## 2 Experimental design, materials, and methods

The data presented in this data article is collected from the simulation results of BSA and the other four competitive algorithms (DE, P*SO, ABS, and FF*) applied to minimise sixteen benchmark functions are presented in three tests [[2], [3], [4], [5]] as follows:

1. Several iterations are needed to minimise a specific function with Nvar variables with the default search space for the population size of 30. Nvars take values of 10, 30, and 60. For each benchmark function, each algorithm is run for 30 times with 2000 iterations for Nvar values of 10, 30, and 60.
2. Several iterations are needed to minimise the functions with two variables for three different sized solution spaces for the population size of 30. For each benchmark function, each algorithm is run for 30 times with 2000 iterations for three different ranges (R1, R2, and R3), where
  (a) R1: [-5, 5]
  (b) R2: [-250, 250]
  (c) R3: [-500, 500]
3. Determining the ratio of successful minimisation of the functions for Tests 1 and 2 is needed to compare the successful rate of BSA with its competitive algorithms.

Table 2, Table 3, Table 4 present basic statistics (Mean: mean-solution, S.D.: standard-deviation of mean-solution, Best: the best solution, Worst: the worst solution, Exec. Time: mean runtime in seconds, No. of succeeds: number of successful minimisation, and No. of Failure: number of failed minimisation) of 30-solutions obtained by BSA, DE, PSO, ABC, and FF in Test 1 to minimise F1–F16 functions with default search space with number of variables 10, 30, and 60 respectively.

In addition, Table 5, Table 6, Table 7 present basic statistics (Mean: mean-solution, S.D.: standard-deviation of mean-solution, Best: the best solution, Worst: the worst solution, Exec. Time: mean runtime in seconds, No. of succeeds: number of successful minimisation, and No. of Failure: number of failed minimisation) of 30-solutions obtained by BSA, DE, PSO, ABC, and FF in Test 2 to minimise F1–F16 functions with default number of variables in three different search spaces R1, R2, and R3 respectively.

In Test 3, the ratio of successful minimisation of the functions for Tests 1 and 2 are presented in Table 8, Table 9.

## Funding

Funding information is not applicable/No funding was received.
